# Femoral anteversion as a potential risk factor for anterior cruciate ligament injuries in athletes

**DOI:** 10.1002/jeo2.70133

**Published:** 2024-12-31

**Authors:** Alireza Mirahmadi, Pooya Hosseini‐Monfared, Maryam Salimi, Arya Kazemi, Naser Ghanbari, Vahid Shameli, Seyed Morteza Kazemi

**Affiliations:** ^1^ Bone Joint and Related Tissues Research Centre Shahid Beheshti University of Medical Sciences Tehran Iran; ^2^ Department of Orthopaedic Surgery Denver Health Medical Centre Denver Colorado USA

**Keywords:** anterior cruciate ligament, athletes, biomechanic, Craig's test, CT scan, femoral anteversion

## Abstract

**Purpose:**

Non‐contact anterior cruciate ligament (ACL) injuries are influenced by the anatomic and biomechanical characteristics of the lower limb. The combination of knee valgus, hip internal rotation and tibial external rotation are important contributors to non‐contact ACL injuries. In this study, we aimed to evaluate the effect of femoral anteversion on the incidence of ACL injuries among athletes.

**Methods:**

A retrospective comparative study was conducted on 137 adult male athletes with high suspicion of ACL injury following a sports‐related injury. The patients were classified into two groups based on the presence of ACL tears: the ‘ACL tear’ and the ‘ACL intact’ groups. The femoral anteversion angle was measured by both a computed tomography (CT) scan using the method described by Hernandez et al. and a physical examination using Craig's test. The association of ACL tears with femoral anteversion angle was evaluated. Femoral anteversion cut‐off values were calculated to find the best margin for a high probability of ACL tearing.

**Results:**

The mean anteversion in patients with ACL tears was found to be higher compared to ACL‐intact patients both in CT scan measures (18.4 ± 5.5 vs. 12.9 ± 6.9, *p* value < 0.001) and physical examination (20.2 ± 5.9 vs. 14.8 ± 7.7, *p* value < 0.001). The correlation analysis revealed an excellent correlation between femoral anteversion measured by CT scan and Craig's test results (*r* = 0.94). Cut‐off values for femoral anteversion measured by CT scan concerning ACL tearing with the highest sensitivity and specificity were 12.7 and 19.0, respectively. The Craig‐measured cut‐off values were 1.5–2° more than the CT scan measurements.

**Conclusion:**

This study reveals a significant correlation between increased femoral anteversion and a higher risk of ACL injury among male athletes. The results of this study aid in designing personalized prevention programmes for non‐contact ACL injuries among athletes.

**Level of Evidence:**

Level III.

**Abbreviations**
ACL: anterior cruciate ligament
BMI: body mass index
CT scan: computed tomography scan
DDH: developmental dysplasia of the hip
ICC: interclass correlation coefficient
MRI: magnetic resonance imaging
OA: osteoarthritis
OR: odds ratio
ROC: receiver operating characteristic
ROM: range of motion

## INTRODUCTION

Anterior cruciate ligament (ACL) injuries are mainly caused by non‐contact mechanisms among athletes, with a higher incidence of injury in women than in men [6, 8, 33, 34]. Among the anatomical and morphological characteristics of the knee, intercondylar notch stenosis, variations in the condylar shape, increased tibial slope, reduced tibial eminence size and reduced ACL size are found to be correlated with a higher incidence of ACL injury [4, 13, 27, 38]. Poor biomechanics of the knee, influenced by trunk positioning and hip kinematics across the frontal, sagittal, and transverse planes, is associated with an increased incidence of non‐contact ACL injuries [11, 31].

Non‐contact ACL injuries occur following landing, sudden deceleration and lateral pivoting manoeuvres without external contact [5]. Indeed, it is widely acknowledged that aberrant hip kinematics and femoral rotational abnormalities are associated with poor knee biomechanics [12, 18, 20]. Adduction and internal rotation of the hip and abduction and external rotation of the tibia cause functional valgus collapse, which is proposed as a potential ACL injury mechanism [19, 22]. Similarly, increased femoral anteversion results in reduced hip congruity, and subsequently, to adopt the decreased congruity, the body may compensate with excessive internal rotation of the hip and functional knee valgus collapse [5, 26].

The femoral neck anteversion angle is defined as the angle between two lines in the axial plane, one passing through the proximal femoral neck region and the other through the distal condylar region. Clinical examinations such as Craig's test or, more reliably, imaging tests like computed tomography (CT) scan or magnetic resonance imaging (MRI) can be used to measure this angle, which indicates how much the femur has been anteverted [14, 25]. Impaired locomotion and altered weight‐bearing during critical growth periods are associated with increased femoral anteversion due to changes in mechanical forces on the developing femur [32].

Gaining a comprehensive understanding of the biomechanically associated risk factors contributing to ACL injury and identifying the proximal femur features that may influence these risks are of utmost importance in developing effective neuromuscular training strategies. The impact of most of the mentioned biomechanical and anatomical parameters has been robustly demonstrated in prior research; however, conflicting evidence regarding the influence of femoral anteversion on ACL injuries exists in the literature. Hence, our study aimed to evaluate the impact of femoral anteversion, measured by both imaging and clinical evaluation, on knee biomechanics and the risk of ACL injuries, specifically in male athletes. We hypothesized that higher femoral anteversion could be a risk factor for non‐contact ACL injury.

## METHODS

A retrospective comparative study was conducted on athletes 18 years of age and older with high suspicion of ACL tears. This study was performed at a referral hospital for sports injuries following institutional review board approval by our institution's Research Medical Ethics Committee (IR.SBMU.RETECH.REC.1399.266), adhering to the principles outlined in the Declaration of Helsinki and institutional ethics guidelines. Written informed consent to use clinical data was obtained from all the patients.

Inclusion criteria (high suspicion of ACL tears) were defined as patients who had a history of knee pain or experienced knee giving away following a sports‐related non‐contact injury and underwent CT scans of the hip and knee according to hospital protocol for athletes, specifically to exclude the presence of minor intraarticular fractures. All patients were evaluated using the Lachman test, pivot shift and MRI examination. Patients who were confirmed to have a complete ACL tear by physical examination and MRI evaluation were classified into the ACL tear group, and patients with an intact ACL without any other knee ligament pathology and bone fracture were considered as the control (ACL intact) group. Exclusion criteria were designated as a previous history of knee injury, a history of the spine, pelvic or lower extremity surgery, a history of hip, pelvic or lower extremity fractures, previous history of ACL injury, previous history of any other ligament and tendon injury in lower limb, paraplegia or hemiplegia, inflammatory arthritis, infectious arthritis, hip or knee OA, a discrepancy of leg length, history of developmental dysplasia of the hip (DDH), connective tissue disorders, and malignancy.

Patients' demographics, including age, gender, weight, height and body mass index (BMI), were recorded for all the participants.

**Figure 1:**
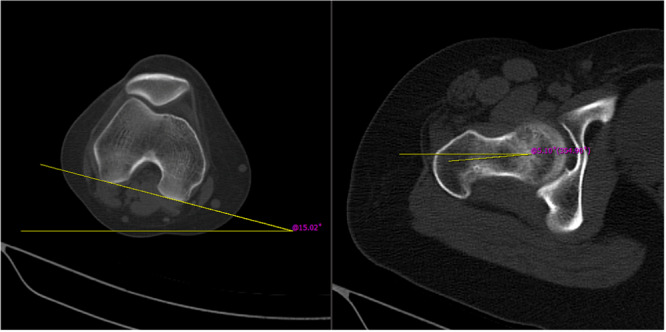
Measurement of femoral anteversion by CT scan. A line drawn from the femoral head's centre to the base of the neck's centre and another line joining the exact posterior sides of the lateral and medial condyles were used to define the femoral anteversion on CT scans (Hernandez et al. method). CT, computed tomography.

A CT scan of the hip and knee was performed on athletes with hip or knee trauma at our hospital to evaluate minor intraarticular fractures. This imaging evaluation follows a predefined protocol established for athletic patients in our institute. Patients were positioned in the supine position with their legs parallel and straight. All radiographs were taken by the same technician and were shown and analyzed on a computer radiography system (Picture Archive and Communication Systems). A line drawn from the femoral head's centre to the base of the neck's centre and another line joining the exact posterior sides of the lateral and medial condyles were used to define the femoral anteversion on CT scans (Figure 1) [16]. Two orthopaedic surgeons conducted all the measurements, and the averages of the two measures were used for data analysis.

**Figure 2:**
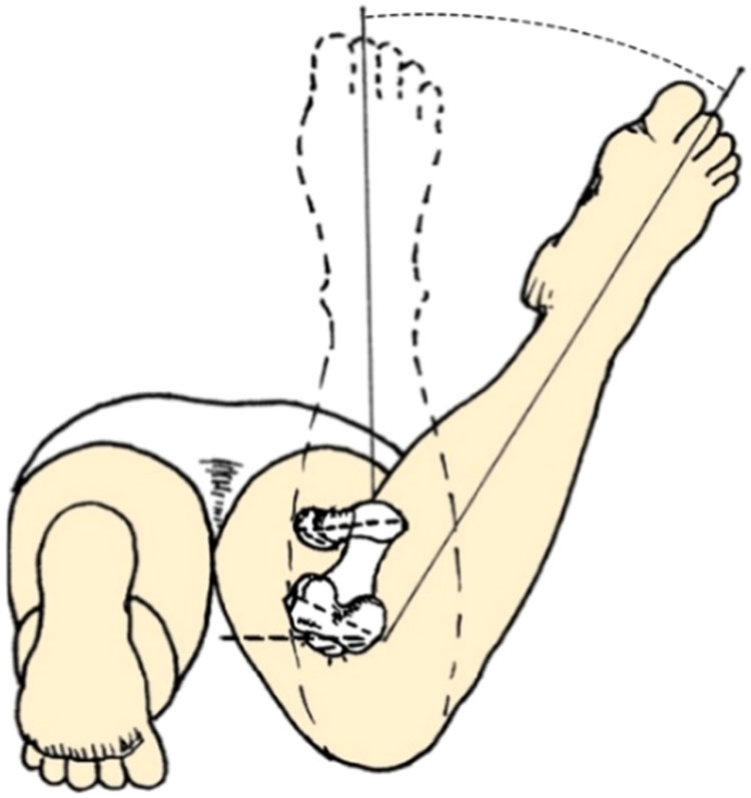
Femoral anteversion measurement using Craig's test. With the patient in the prone position and the knee flexed to 90°, the examiner palpated the greater trochanter and passively rotated the hip to position the trochanter at its most lateral prominence. The angle between the true vertical axis and the tibial shaft was then measured using a goniometer.

Also, the femoral anteversion was assessed by an experienced orthopaedic surgeon using Craig's test, who was unaware of CT scan measures. In the prone position, with the patient's knee flexed to 90°, the examiner palpated the greater trochanter and passively rotated the hip until the most prominent portion of the greater trochanter was in its most lateral position. The angle between the real vertical and the shaft of the tibia was then measured using a goniometer (Figure 2) [30].

The femoral anteversion evaluated by Craig's test was compared to the femoral anteversion measured via CT scan, and the correlation between the two measurements was determined using the Pearson correlation coefficient. Also, A two‐way mixed effect interclass correlation coefficient (ICC) was used to assess the level of agreement between the two methods of measuring femoral anteversion in patients with ACL injury. Values less than 0.5 indicate poor agreement, values between 0.5 and 0.75 indicate moderate agreement, values between 0.75 and 0.9 indicate good agreement, and values greater than 0.90 indicate excellent agreement [29].

All patients referred to our centre who met the study's inclusion and exclusion criteria and had the necessary imaging were included in this research. Based on the sample size of our study, a power analysis conducted with reference to prior studies [2, 7], using an *α* level of 0.05, demonstrated a statistical power exceeding 95% for the primary objectives of our investigation.

The Kolmogorov–Smirnov test was used to assess the normality of distributions. Mean values, standard deviations and 95% confidence intervals (CIs) were calculated. An independent two‐sample *t* test was used to compare the anteversion between the ACL‐injured patients and the control group. Pearson correlation coefficients were used to determine the concurrent validity of the physical examinations (Craig test) with regard to femoral anteversion and the angle as measured on the CT scans. The correlation was considered poor (0.00–0.20), fair (0.21–0.40), moderate (0.41–0.60), good (0.61–0.80) or excellent (0.81–1.00) [29]. In order to estimate the ICC and their 95% CIs, we employed a mean‐rating (*k* = 2), consistency, two‐way mixed‐effects model. CT scan result was predicted with a linear regression model. Femoral anteversion cut‐off values were calculated with proper sensitivity and specificity with receiver operating characteristic (ROC) curve analysis to find the best margin for a high probability of ACL tearing. SPSS statistics (version 29; SPSS) was used for the statistical analysis, and statistical significance was accepted for *p* values of <0.05.

## RESULTS

A total of 137 active male athletes aged 18–35 with previous semi‐professional or professional sports activity were evaluated for knee pain or the experience of giving way at a tertiary referral hospital. Among the patients, 85 patients had positive Lachman and anterior drawer tests and had their ACL tears confirmed by MRI and subsequently underwent ACL reconstruction. The remaining 52 patients, who showed no significant knee pathology in clinical and radiological evaluations, were designated as the control group for comparison. Included patients were volleyball, basketball, and soccer players. The mean age and BMI of the patients were not significantly different between the ACL tear and control group (*p* = n.s.). The demographic characteristics of the participants are summarized in Table 1.

**Table 1:** Demographic features of the patients.

	Total	ACL tear group	Control group	
Variable	*n* = 137 (100%)	*n* = 85 (62%)	*n* = 52 (38%)	*p*
Age (years)	24.4 ± 6.4	25.0 ± 4.3	22.9 ± 3.2	n.s.
BMI (kg/m^2^)	23.4 ± 3.9	24.2 ± 3.9	23.1 ± 3.1	n.s.

Abbreviation: BMI, body mass index.

### Femoral anteversion angle and ACL injury

The patients with ACL tear demonstrated a significantly higher mean femoral anteversion angle, as measured by both CT and Craig tests, compared to the Control group (*p* < 0.001) (Table 2). There was no significant correlation between anteversion degree and patients' demographic characteristics (*p* = n.s.).

**Table 2:** Femoral anteversion angles (mean ± SD).

Femoral anteversion angle	Total	ACL tear group	Control group	*p*
By CT scan	17.1 ± 6.3	18.4 ± 5.5	12.9 ± 6.9	**<0.001** *
By Craig's test	18.9 ± 6.8	20.2 ± 5.9	14.8 ± 7.7	**<0.001** *

Abbreviations: ACL, anterior cruciate ligament; CT, computed tomography; SD, standard deviation.

* *p* value < 0.05.

### Agreement of Craig's test and CT scan for determining femoral anteversion

**Figure 3:**
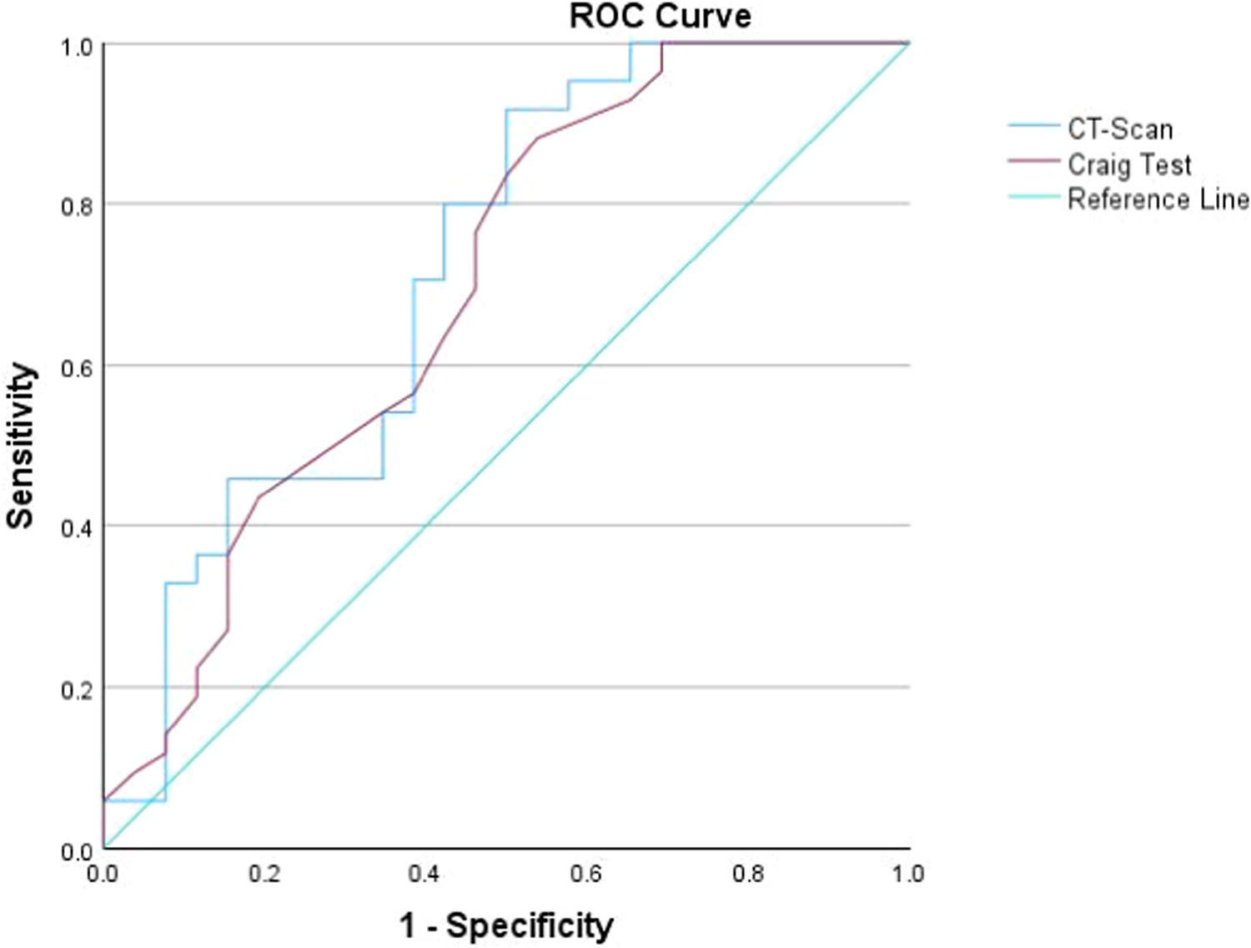
ROC curve analysis for determining femoral anteversion cut‐off values associated with ACL tearing. ACL, anterior cruciate ligament; CT, computed tomography; ROC, receiver operating characteristic.

Femoral anteversion measured by CT scan was significantly lower than that of the Craig test (*p* < 0.001, 1.8 ± 2.3, range from −7.5 to 10.5), and there was a significant correlation between femoral anteversion measured by CT scan and Craig test (r = 0.94) which is considered as an excellent correlation (Figure 3) [30]. A two‐way mixed effect ICC of Craig's test and CT scan was 0.97 (95% CI = 0.95–0.98), determining excellent agreement.

Multiple linear regression was used to predict the femoral anteversion (confirmed by CT scan as the gold standard) based on patients' physical examination (Craig's test) and demographic features. A significant regression equation was found (*R* ^2^ = 0.79, *p* < 0.001, *F*(3.7) = 95.6). Patients' Craig test was the only statistically significant predictor (*p* < 0.001, *B* = 0.72) to predict exact femoral anteversion, measured by CT scan.

### Femoral anteversion cut‐off values for ACL tearing

ROC curve analysis was conducted to calculate the femoral anteversion cut‐off values for ACL tearing (Figure 3). Based on Youden's index, the best cut‐offs with acceptable sensitivity and specificity were considered for both the CT scan and Craig tests. Femoral anteversion cut‐off values with high specificity were determined as 19.0 and 20.5 for the CT scan and physical examination, respectively (Table 3). As the odds ratios (ORs) showed, the possibility of ACL tearing in the patients with femoral anteversion lower than the calculated cut‐off is significantly lower than in other patients with femoral anteversion higher than the cut‐off. Also, comparing these suggested cut‐offs with 15°, which is the most commonly reported upper anteversion value for the normal population, femoral anteversion 15° has weaker OR, sensitivity, and specificity in predicting ACL tears.

**Table 3:** ROC curve analysis for femoral anteversion.

	Cut‐off Value	Sensitivity (%)	Specificity (%)	*p*	AUC (%)	OR/95% CI
CT scan	12.7	92	50	**0.001** *	70	0.09/0.03–0.27
	19.0	46	85	**0.001** *	70	0.21/0.07–0.68
Craig test	14.5	84	50	**0.003** *	72	0.19/0.08–0.51
	20.5	44	81	**0.003** *	72	0.31/0.11–0.89

Abbreviations: AUC, area under the curve; CI, confidence interval; CT, computed tomography; OR, odds ratio; ROC, receiver operating characteristic.

* *p* value < 0.05.

## DISCUSSION

The most important finding of this study was that the femoral anteversion angle, as measured by CT scan and Craig's test, was significantly higher in the ACL tear group. This suggests that increased femoral anteversion is associated with a higher risk of ACL injury among male athletes. The femoral anteversion high specific cut‐off values of 19° for a CT scan and 20.5° for a physical examination were determined by ROC analysis to predict a higher risk of ACL injury [32]. Though the cut‐off of 12.7° for the CT scan and 14.5° for Craig's test had higher sensitivity, we prioritized specificity to use it to find the best margin for a high probability of future ACL tearing in athletes.

In a study using imaging modalities for measuring the femoral anteversion, Alplay et al. conducted a retrospective study on 2344 patients with a history of non‐contact ACL injury and evaluated the femoral anteversion of these patients with MRI [2]. They found that femoral anteversion and infratrochanteric torsion were higher in patients with ACL injuries. However, supratrochanteric torsion was not different between ACL‐intact and ACL‐deficient groups [2]. Our findings also confirmed that excessive femoral anteversion measured by imaging (CT scan) correlated with a higher risk of ACL injury than the control group. Furthermore, the ROC curve analysis of our study showed that femoral anteversion higher than 12.7° and 19° in CT scan has a high sensitivity and specificity for predicting ACL injury, respectively.

Non‐contact ACL injuries are known to occur following complex and multiplanar load states of the knee. Kaneko et al. conducted a kinematic analysis involving 16 female participants upon single‐leg landings. They compared the kinematic analysis and muscle activity results between the participants with high and low femoral anteversion measured by Craig's test. The motion analysis demonstrated that higher femoral anteversion results in lower hip flexion angle, higher knee valgus alignment and greater rectus femoris muscle activity, leading to anterior tibial displacement [21]. The observed mechanism of their study highlights the potential risk of femoral anteversion for ACL injury in athletes. Excessive femoral anteversion angle is associated with reduced hip joint congruity, which finally leads the body to compensate with excessive hip internal rotation. This results in functional knee valgus collapse, a combination of knee valgus, hip internal rotation and tibial external rotation, which is the proposed cause of a higher rate of ACL injury among people with higher anteversion angle [17].

A study on 53 male athletes with complete ACL tears was conducted by Amraee et al. to evaluate the lower extremity measures in this population [3]. They measured the unilateral navicular drop, the ankle dorsiflexion range of motion (ROM), internal tibia torsion, knee genu recurvatum, quadriceps angle, hip internal and external rotation ROM, and hip anteversion in patients with complete ACL injury and compared with the accepted standard values. Their study demonstrated that increased hip anteversion, decreased hip internal rotation range, and decreased ankle dorsiflexion were predictors of ACL injury. The multiple logistic regression analysis revealed that an increase in femoral anteversion by 1° would increase the likelihood of sustaining an ACL injury by 1.78 (*p* < 0.05). In their study, they did not have any control group as a comparison, and they measured the femoral anteversion only through physical examination and Craig's test [3]. Our findings aligned with previous studies demonstrating that excessive femoral anteversion measured by both Craig's test and CT scan correlated with a higher risk of ACL injury than the control group.

In contrast, case–control studies of females with ACL injury demonstrated that the femoral anteversion was not significantly different in patients with ACL injury compared to those without ACL injury [9, 23]. The small sample sizes of the mentioned studies and the fact that only female patients were included may account for the different findings observed in these two studies compared to our results. Furthermore, previous studies have indicated a rise in the occurrence of ACL injuries among female athletes, possibly linked to a slightly greater anteversion in females. We specifically evaluated 137 male athletes who have lower femoral anteversion angles compared to females based on previous studies [15, 28].

Most of the mentioned studies evaluated the femoral anteversion only by physical examination, which is based on the palpation of the greater trochanter, which has a potential for palpation error due to obesity or scarring in the region [35]. Furthermore, knee joint laxity affects the measured femoral anteversion angle by Craig's test and causes the measured angles to be estimated higher than by imaging methods [10, 36, 39]. In this study, we measured femoral anteversion by both CT scans and physical examination, and the results showed a proper correlation between these two measurement methods. The high correlation of Craig's test with the CT scan in our study may be because of the lack of disruptive factors in the patients we included, such as obesity, scars in the region, and joint laxity.

Botser et al. compared the femoral anteversion measurements by physical examination with imaging studies, including MRI and CT scans on 129 hips [7]. They concluded that excessive femoral anteversion should not rely on hip joint ROM, which is evaluated only by physical examination. Their findings indicated that while femoral anteversion assessments through CT scans and MRI exhibited a strong correlation, notable inconsistencies were noted in the exact anteversion angle, suggesting that these modalities may not be easily substituted. Furthermore, the study revealed that CT scans yielded higher interobserver reliability when conducting anteversion measurements. It was mentioned that measuring femoral anteversion has different values according to physical examination compared to CT scans or MRI measurements [7, 30, 37]. Our findings demonstrated that the femoral anteversion angles measured by CT scan were lower than those measured by Craig's test. However, we observed a significant and robust correlation between the femoral anteversion angles measured by these two methods.

Similar to the other retrospective studies, this study had several limitations. The study's retrospective nature leads to uncontrolled conditions that may increase the biases of the result. Our study only demonstrated an association between femoral anteversion and ACL tears, not a causal relationship. So, patient selection and prevention programmes are more recommended than direct intervention to adjust femoral anteversion. Since all the patients were male athletes, the results should be carefully interpreted, especially for female athletes and generalizability. Also, the effects of other alignment variables related to the biomechanics of the lower limb were not considered in this study. Prospectively designed studies with larger sample sizes, both biologically female and male, and considering other influential variables are required to evaluate the correlation between lower limb alignment and ACL injuries.

In this study, we evaluated the effect of excessive femoral anteversion, which was determined by physical examination and CT scan on the incidence of ACL injuries among male athletes. Our study's design is novel, and patients in the control group are athletes similar to the ACL‐injured group with similar trauma but without ACL tears, which no previous studies have compared before. This study's calculated cut‐off values for femoral anteversion can be particularly useful in identifying individuals at risk for ACL injury [1]. Since this is a non‐modifiable risk factor, ACL injury prevention training could be an effective preventive measure for patients with higher femoral anteversion. The insights obtained from this study provide healthcare professionals with the essential knowledge to recommend ACL prevention programmes that have been shown to be effective in reducing ACL injury rates, such as sport technique modification, neuromuscular control, proper landing techniques and plyometric training, for high‐risk patients [1, 24]. The findings of this study can aid in designing personalized ACL prevention programmes other than femoral osteotomy for patients who are more susceptible to ACL injuries.

## CONCLUSION

Our study has highlighted the correlation between femoral anteversion and ACL injury in young male athletes. The mean anteversion in both CT scans and physical examinations of patients with ACL tears was higher than in ACL‐intact patients. The findings of this study can aid in predicting and preventing ACL injuries in professional athletes.

## AUTHOR CONTRIBUTIONS

Alireza Mirahmadi, Maryam Salimi, Naser Ghanbari and Seyed Morteza Kazemi conceptualized the study. Pooya Hosseini‐Monfared, Maryam Salimi, Vahid Shameli and Arya Kazemi performed the investigation. Alireza Mirahmadi and Pooya Hosseini‐Monfared carried out the analysis. Pooya Hosseini‐Monfared, Alireza Mirahmadi, Maryam Salimi and Arya Kazemi drafted and Naser Ghanbari, Seyed Morteza Kazemi and Vahid Shameli revised the paper. All authors read and approved the final manuscript.

## CONFLICT OF INTEREST STATEMENT

The authors declare no conflicts of interest.

## ETHICS STATEMENT

The study received ethical approval from the institutional review board (IR.SBMU.RETECH.REC.1399.266) ensuring compliance with ethical guidelines and transparency in the research process.

## Data Availability

The data used and analyzed during the current study are available from the corresponding author upon reasonable request.

## References

[1] Alentorn‐Geli, E. , Mendiguchía, J. , Samuelsson, K. , Musahl, V. , Karlsson, J. , Cugat, R. et al. (2014) Prevention of non‐contact anterior cruciate ligament injuries in sports. Part II: systematic review of the effectiveness of prevention programmes in male athletes. Knee Surgery, Sports Traumatology, Arthroscopy, 22, 16–25. Available from: 10.1007/s00167-013-2739-x 24162718

[2] Alpay, Y. , Ezici, A. , Kurk, M.B. , Ozyalvac, O.N. , Akpinar, E. & Bayhan, A.I. (2020) Increased femoral anteversion related to infratrochanteric femoral torsion is associated with ACL rupture. Knee Surgery, Sports Traumatology, Arthroscopy, 28, 2567–2571. Available from: 10.1007/s00167-020-05874-0 32030504

[3] Amraee, D. , Alizadeh, M.H. , Minoonejhad, H. , Razi, M. & Amraee, G.H. (2017) Predictor factors for lower extremity malalignment and non‐contact anterior cruciate ligament injuries in male athletes. Knee Surgery, Sports Traumatology, Arthroscopy, 25, 1625–1631. Available from: 10.1007/s00167-015-3926-8 26704803

[4] Bayer, S. , Meredith, S.J. , Wilson, K.W. , de Sa, D. , Pauyo, T. , Byrne, K. et al. (2020) Knee morphological risk factors for anterior cruciate ligament injury: a systematic review. Journal of Bone and Joint Surgery, 102, 703–718. Available from: 10.2106/JBJS.19.00535 31977822

[5] Beaulieu, M.L. , Oh, Y.K. , Bedi, A. , Ashton‐Miller, J.A. & Wojtys, E.M. (2014) Does limited internal femoral rotation increase peak anterior cruciate ligament strain during a simulated pivot landing? The American Journal of Sports Medicine, 42, 2955–2963. Available from: 10.1177/0363546514549446 25245132 PMC6380493

[6] Boden, B.P. , Dean, G.S. , Feagin, J.A. & Garrett, W.E. (2000) Mechanisms of anterior cruciate ligament injury. Orthopedics, 23, 573–578. Available from: 10.3928/0147-7447-20000601-15 10875418

[7] Botser, I.B. , Ozoude, G.C. , Martin, D.E. , Siddiqi, A.J. , Kuppuswami, S. & Domb, B.G. (2012) Femoral anteversion in the hip: comparison of measurement by computed tomography, magnetic resonance imaging, and physical examination. Arthroscopy: The Journal of Arthroscopic & Related Surgery, 28, 619–627. Available from: 10.1016/j.arthro.2011.10.021 22301362

[8] Chia, L. , De Oliveira Silva, D. , Whalan, M. , McKay, M.J. , Sullivan, J. , Fuller, C.W. et al. (2022) Non‐contact anterior cruciate ligament injury epidemiology in team‐ball sports: a systematic review with meta‐analysis by sex, age, sport, participation level, and exposure type. Sports Medicine, 52, 2447–2467. Available from: 10.1007/s40279-022-01697-w 35622227 PMC9136558

[9] Daneshmandi, H. , Saki, F. , Daneshmandi, L. & Daneshmandi, M. (2012) Lower extremity alignment in female athletes with ACL reconstruction. Medical Sport, 65, 211–221.

[10] Davids, J.R. , Benfanti, P. , Blackhurst, D.W. & Allen, B.L. (2002) Assessment of femoral anteversion in children with cerebral palsy: accuracy of the trochanteric prominence angle test. Journal of Pediatric Orthopaedics, 22, 173–178. Available from: 10.1097/01241398-200203000-00007 11856924

[11] Donelon, T.A. , Dos'Santos, T. , Pitchers, G. , Brown, M. & Jones, P.A. (2020) Biomechanical determinants of knee joint loads associated with increased anterior cruciate ligament loading during cutting: a systematic review and technical framework. Sports Medicine – Open, 6, 53. Available from: 10.1186/s40798-020-00276-5 33136207 PMC7606399

[12] Flury, A. , Hodel, S. , Hasler, J. , Hooman, E. , Fucentese, S.F. & Vlachopoulos, L. (2022) The winking sign is an indicator for increased femorotibial rotation in patients with recurrent patellar instability. Knee Surgery, Sports Traumatology, Arthroscopy, 30, 3651–3658. Available from: 10.1007/s00167-022-06971-y 35438307 PMC9568440

[13] Griffin, L.Y. , Agel, J. , Albohm, M.J. , Arendt, E.A. , Dick, R.W. , Garrett, W.E. et al. (2000) Noncontact anterior cruciate ligament injuries: risk factors and prevention strategies. Journal of the American Academy of Orthopaedic Surgeons, 8, 141–150. Available from: 10.5435/00124635-200005000-00001 10874221

[14] Grünwald, L. , Histing, T. , Springer, F. & Keller, G. (2023) MRI‐based torsion measurement of the lower limb is a reliable and valid alternative for CT measurement: a prospective study. Knee Surgery, Sports Traumatology, Arthroscopy, 31, 4903–4909. Available from: 10.1007/s00167-023-07533-6 37589766 PMC10598136

[15] Hartel, M.J. , Petersik, A. , Schmidt, A. , Kendoff, D. , Nüchtern, J. , Rueger, J.M. et al. (2016) Determination of femoral neck angle and torsion angle utilizing a novel three‐dimensional modeling and analytical technology based on CT datasets. PLoS One, 11, e0149480. Available from: 10.1371/journal.pone.0149480 26933877 PMC4775021

[16] Hernandez, R. , Tachdjian, M. , Poznanski, A. & Dias, L. (1981) CT determination of femoral torsion. American Journal of Roentgenology, 137, 97–101. Available from: 10.2214/ajr.137.1.97 6787898

[17] Hogg, J.A. , Waxman, J.P. & Shultz, S.J. (2022) Examining the effects of femoral anteversion and passive hip rotation on ACL injury and knee biomechanics: a systematic review and meta‐analysis. Journal of Experimental Orthopaedics, 9, 40. Available from: 10.1186/s40634-022-00479-7 35513749 PMC9072613

[18] Howard, J. (2011) Structure, sex, and strength and knee and hip kine. 10.4085/1062-6050-46.4.376 PMC3419149 21944069

[19] Ireland, M.L. (1999) Anterior cruciate ligament injury in female athletes: epidemiology. Journal of Athletic Training, 34, 150–154. 16558558 PMC1322904

[20] Jacobs, C.A. , Uhl, T.L. , Mattacola, C.G. , Shapiro, R. & Rayens, W.S. (2007) Hip abductor function and lower extremity landing kinematics: sex differences. Journal of Athletic Training, 42, 76–83. 17597947 PMC1896084

[21] Kaneko, M. & Sakuraba, K. (2013) Association between femoral anteversion and lower extremity posture upon single‐leg landing: implications for anterior cruciate ligament injury. Journal of Physical Therapy Science, 25, 1213–1217. Available from: 10.1589/jpts.25.1213 24259760 PMC3820182

[22] Koga, H. , Nakamae, A. , Shima, Y. , Iwasa, J. , Myklebust, G. , Engebretsen, L. et al. (2010) Mechanisms for noncontact anterior cruciate ligament injuries: knee joint kinematics in 10 injury situations from female team handball and basketball. The American Journal of Sports Medicine, 38, 2218–2225. Available from: 10.1177/0363546510373570 20595545

[23] Kramer, L.C. , Denegar, C.R. , Buckley, W.E. & Hertel, J. (2007) Factors associated with anterior cruciate ligament injury: history in female athletes. The Journal of Sports Medicine and Physical Fitness, 47, 446–454. 18091686

[24] Mandelbaum, B.R. , Silvers, H.J. , Watanabe, D.S. , Knarr, J.F. , Thomas, S.D. , Griffin, L.Y. et al. (2005) Effectiveness of a neuromuscular and proprioceptive training program in preventing anterior cruciate ligament injuries in female athletes: 2‐year follow‐up. The American Journal of Sports Medicine, 33, 1003–1010. Available from: 10.1177/0363546504272261 15888716

[25] Muhamad, A.R. , Freitas, J.M. , Bomar, J.D. , Dwek, J. & Hosalkar, H.S. (2012) CT and MRI lower extremity torsional profile studies: measurement reproducibility. Journal of Children's Orthopaedics, 6, 391–396. Available from: 10.1007/s11832-012-0434-y PMC3468734 24082954

[26] Nguyen, A.D. , Shultz, S.J. & Schmitz, R.J. (2015) Landing biomechanics in participants with different static lower extremity alignment profiles. Journal of Athletic Training, 50, 498–507. Available from: 10.4085/1062-6050-49.6.03 25658815 PMC4560011

[27] Nukuto, K. , Gale, T. , Yamamoto, T. , Musahl, V. & Anderst, W. (2023) Bone morphology features associated with knee kinematics may not be predictive of ACL elongation during high‐demand activities. Knee Surgery, Sports Traumatology, Arthroscopy, 31, 5096–5103. Available from: 10.1007/s00167-023-07560-3 37728761

[28] Pierrepont, J.W. , Marel, E. , Baré, J.V. , Walter, L.R. , Stambouzou, C.Z. , Solomon, M.I. et al. (2020) Variation in femoral anteversion in patients requiring total hip replacement. HIP International, 30, 281–287. Available from: 10.1177/1120700019848088 31084219

[29] Portney, L.G. (2020) Foundations of clinical research: applications to evidence‐based practice. FA Davis.

[30] Ruwe, P.A. , Gage, J.R. , Ozonoff, M.B. & DeLuca, P.A. (1992) Clinical determination of femoral anteversion. A comparison with established techniques. The Journal of Bone & Joint Surgery, 74, 820–830. Available from: 10.2106/00004623-199274060-00003 1634572

[31] Schaver, A.L. , Grezda, K. , Willey, M.C. & Westermann, R.W. (2021) Radiographic cam morphology of the hip may be associated with ACL injury of the knee: a case‐control study. Arthroscopy, Sports Medicine, and Rehabilitation, 3, e1165–e1170. Available from: 10.1016/j.asmr.2021.05.004 34430897 PMC8365193

[32] Scorcelletti, M. , Reeves, N.D. , Rittweger, J. & Ireland, A. (2020) Femoral anteversion: significance and measurement. Journal of Anatomy, 237, 811–826. Available from: 10.1111/joa.13249 32579722 PMC7542196

[33] Silvers, H.J. & Mandelbaum, B.R. (2007) Prevention of anterior cruciate ligament injury in the female athlete. British Journal of Sports Medicine, 41, i52. Available from: 10.1136/bjsm.2007.037200 17609222 PMC2465242

[34] Sutton, K.M. & Bullock, J.M. (2013) Anterior cruciate ligament rupture: differences between males and females. Journal of the American Academy of Orthopaedic Surgeons, 21, 41–50. Available from: 10.5435/JAAOS-21-01-41 23281470

[35] Tamari, K. , Tinley, P. , Briffa, K. & Breidahl, W. (2005) Validity and reliability of existing and modified clinical methods of measuring femoral and tibiofibular torsion in healthy subjects: use of different reference axes may improve reliability. Clinical Anatomy, 18, 46–55. Available from: 10.1002/ca.20050 15597368

[36] Testa, R. , Chouteau, J. , Philippot, R. , Cheze, L. , Fessy, M. & Moyen, B. (2010) In vitro analysis of varus‐valgus laxity of the knee joint: comparison of clinical evaluation with measurements using a reference motion analysis system. IRBM, 31, 302–308. Available from: 10.1016/j.irbm.2010.10.005

[37] Uota, S. , Morikita, I. & Shimokochi, Y. (2019) Validity and clinical significance of a clinical method to measure femoral anteversion. The Journal of Sports Medicine and Physical Fitness, 59, 1908–1914. Available from: 10.23736/S0022-4707.19.09733-0 31215203

[38] Yahagi, Y. , Gale, T. , Nukuto, K. , Irrgang, J. , Musahl, V. & Anderst, W. (2024) Tibial spine volume is smaller in ACL‐injured athletes compared to healthy athletes. Knee Surgery, Sports Traumatology, Arthroscopy, 32, 1370–1375. Available from: 10.1002/ksa.12161 38529659

[39] Yoon, T.L. , Park, K.M. , Choi, S.A. , Lee, J.H. , Jeong, H.J. & Cynn, H.S. (2014) A comparison of the reliability of the trochanteric prominence angle test and the alternative method in healthy subjects. Manual Therapy, 19, 97–101. Available from: 10.1016/j.math.2013.07.011 24035201

